# The 9-Phenyl-9-fluorenyl Group for Nitrogen Protection in Enantiospecific Synthesis

**DOI:** 10.3390/molecules15096512

**Published:** 2010-09-17

**Authors:** Essi J. Karppanen, Ari M. P. Koskinen

**Affiliations:** Laboratory of Organic Chemistry, Department of Chemistry, Aalto University, School of Science and Technology, PO Box 16100, Kemistintie 1, FI-00076 Aalto, Finland; Email: essi.karppanen@tkk.fi

**Keywords:** phenylfluorenyl, amino acid, nitrogen protecting group, enantiospecific, enantiopure

## Abstract

One of the biggest challenges in asymmetric synthesis is to prevent racemization of enantiopure starting materials. However, at least some of the enantiopurity is lost in most of the existing reactions used in synthetic organic chemistry. This translates into unnecessary material losses. Naturally enantiopure proteinogenic amino acids that can be transformed into many useful intermediates in drug syntheses, for example, are especially vulnerable to this. The phenylfluoren-9-yl (Pf) group, a relatively rarely used protecting group, has proven to be able to prevent racemization in α-amino compounds. This review article showcases the use of Pf-protected amino acid derivatives in enantiospecific synthesis.

## 1. Introduction

Of the 20 natural proteinogenic amino acids, 19 are chiral, and being readily commercially available, they are potentially useful educts for enantiospecific synthesis. Typically the nitrogen atom needs to be protected to allow further manipulations of the carboxylic acid center. Common nitrogen protecting groups include removable alkyl groups such as benzyl and substituted benzyl groups, or more frequently carbamates such as tert-butyl, benzyl or fluorenylmethyl carbamates (Boc, Cbz and Fmoc, respectively). Common nitrogen protecting groups do not fare well in guarding the enantiomeric purity of α-chiral amino carbonyl compounds, because they fail to shield the acidic α-proton from removal. Surprisingly little attention has been paid to the fact that most of the chemistry emanating from these valuable chiral pool educts often leads to the destruction of significant amounts of the chiral information and even under carefully controlled conditions some erosion of enantiopurity is observed. The 9-phenylfluoren-9-yl (Pf) group has proven to be an outstanding choice for protecting the α-center of the amino acid derivatives against epimerization and some 250 papers and patents have been published on the chemistry of the Pf group acting as a protecting group on nitrogen. Rather than offering a comprehensive review, we have selected representative references to highlight the pros and cons of the chemistry based on Pf protection. 

## 2. The Phenylfluorenyl Group

### 2.1. Generalities

Structurally the phenylfluorenyl (Pf) group resembles the trityl protecting group. Trityl groups were first used to protect amines instead of *t*-butyl and phenylmethyl carbamates (Fmoc, Boc, Cbz) in order to protect the α-center, and *N*-trityl amino compounds were observed to retain their enantiopurity with >90% ee [1]. However, the resulting tritylamines are acid labile, thus rendering them of limited use, and alternative protecting groups of similar size were needed. The Pf group, known to be solvolytically >6,000 times more acid stable than trityl [2] entered the picture in the 1980s [3].

**Figure 1:**
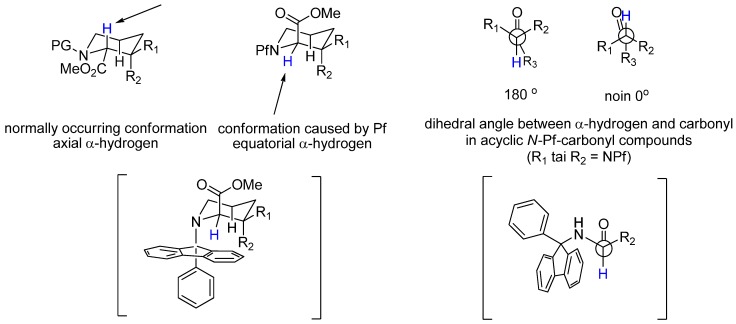
Conformational analysis of cyclic and acyclic compounds.

**Figure 2:**
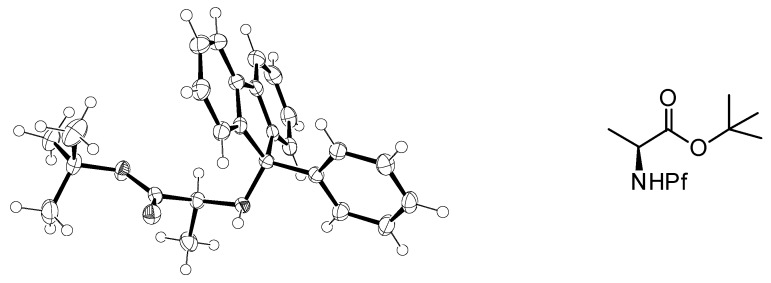
Crystal structure of *N*-Pf alanine *tert*-butyl ester [6].

The strength of the Pf group lies in its sterically demanding size that protects the α-center. It has been calculated that in an *N*-Pf α-amino acid derivative, the steric bulk of the Pf group forces the compound to adopt a conformation in which the dihedral angle between the carbonyl group and the α-hydrogen is ~ 0° or 180° [4]. In cyclic compounds the Pf group drives the α-hydrogen into an equatorial position [5]. (Figure 1 and Figure 2)

Not only the steric bulk of the Pf group, but also stereoelectronics may play a role in its ability to protect the α-center. For example in the case of esters, this could be further explained as follows: the most favorable conformation for deprotonation is one in which the α-hydrogen-carbon bond lies in a plane orthogonal to the plane of the carbonyl system, allowing for maximum orbital overlap as the α-carbon rehybridizes from sp^3^ to sp^2^. However, the α-proton and the carbonyl group in *N*-Pf-protected esters are nearly coplanar. Thus the rate of α-deprotonation would be stereoelectronically retarded in any ester for which such a conformation is highly favored.

According to crystal structure analyses the α-ester group and the fluorenyl ring are in close contact. This is strongly supported by the fact that the proton NMR shows a methyl ester resonance at 2.92 ppm (an unusually upfield chemical shift). So, achieving a stereoelectronically favorable conformation during deprotonation would require the ester group to rotate into the region of space occupied by the Pf group, resulting in allylic strain in the enolate-like transition state. This has been observed in the context with the alkylation of *N*-Pf-L-glutamate [7].

**Scheme 1:**
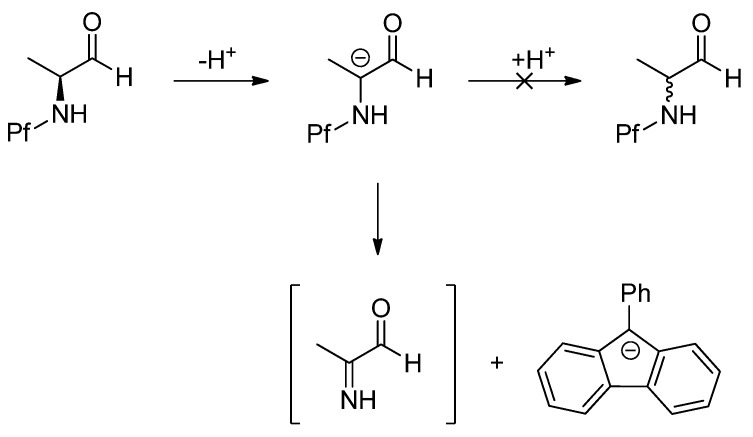
The decomposition of *N*-Pf amino aldehyde in basic conditions.

The racemization of *N*-Pf-compounds could be considered very unlikely after further investigating the stability in basic conditions. In strongly basic conditions the Pf anion can act as a leaving group giving rise to an imine (Scheme 1). This elimination occurs faster/easier than the inversion and reprotonation of the anion [5].

### 2.2. Phenylfluorenylation

**Scheme 2:**
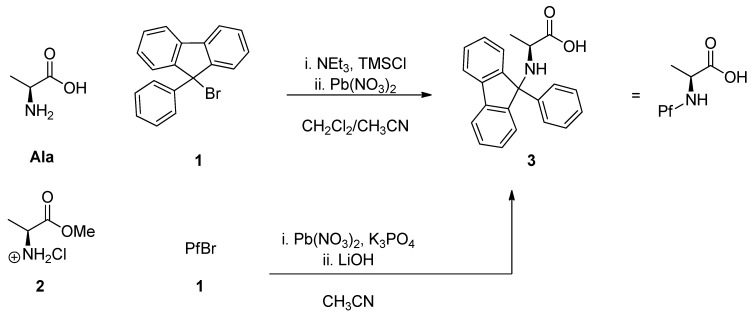
Traditional phenylfluorenylation [8].

Phenylfluorenylation was originally carried out by modifying and applying the existing method for making *N*-trityl compounds [3], which is still the most widely used. This traditional method utilizes 9-bromo-9-phenylfluorene (PfBr, **1**). The protection is based on improving Br’s role as a leaving group with halogenophilic lead nitrate. The amino acid can be used free as such, when it has to be temporarily protected with TMSCl. It is also possible to use the corresponding methyl ester HCl salt **2**. (Scheme 2) Phenylfluorenylation is slow, taking several days, but the reaction is robust and the yields are generally good [8].

### 2.3. Cleavage of Pf from nitrogen

The Pf group is usually removed by hydrogenolysis [9] and solvolysis under strongly acidic conditions [5]. It can also be removed by lithium or sodium in ammonia solution [10], by TMSOTf in the presence of triethyl silane [11] or by iodine in methanol [12].

### 2.4. Phenylfluorenyl cation

Phenyfluorenyl cations have been made from fluorenol with strong acids such as sulphonic or triflic acid. In solution the cations are deep red and with an absorption maximum at λ_max_ = 494 nm. Quenching the cation with methanol forms Pf methyl ether as the main product [13].

## 3. The Phenylfluorenyl Group in Synthesis

The Pf group has been used as a protecting group for nitrogen atoms exclusively in amino acids. It maintains the configurational integrity of the α-amino compounds during different kinds of C-C forming reactions. Enolization and alkylation [14], Wittig and aldol reactions [15], as well as Grignard addition [14] of *N*-Pf-amino carbonyl compounds are examples of reactions where the enantiomeric purity is preserved. Actually this applies to all examples presented in this review, although it is not mentioned in the form of ee’s (>99.9%) later on.

### 3.1. Amino aldehydes

Carbamate protected amino aldehydes are versatile intermediates in the synthesis of polyfunctional amino acids, peptide analogues, sphingolipids and aminosugars. They are configurationally unstable and racemize, even upon rapid chromatography on silica gel [16]. The enantiomeric purity is usually not maintained in nucleophilic additions. Trityl-protected amino aldehydes are less prone to racemization than carbamate protected ones, but they are extremely acid labile, even under mildly acidic conditions. *N*-Pf-Amino aldehydes are configurationally stable when subjected to both additions and flash chromatography [5].

**Scheme 3:**
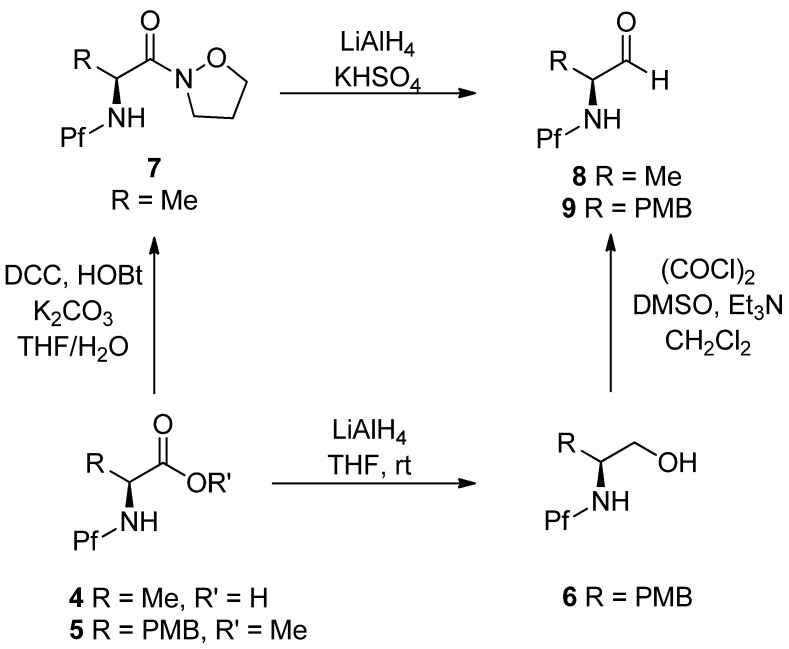
Preparation of amino aldehydes.

*N*-Pf-Amino aldehydes can be prepared from amino acids by reducing the ester to the alcohol with LiAlH_4_ and then oxidizing it to aldehyde by Swern oxidation [17]. Another option is to form an isoxazolidide and reduce it to the aldehyde with LiAlH_4_ [5]. Preparation of aldehydes is outlined in Scheme 3.

**Scheme 4:**
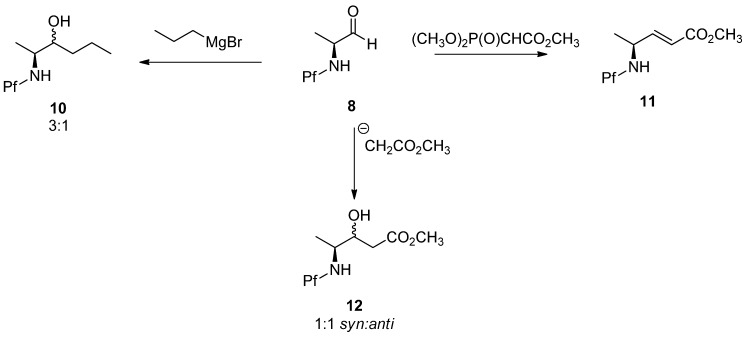
Grignard, Wittig and aldol addition of *N*-Pf-amino aldehydes.

Grignard, Wittig and aldol additions of *N*-Pf-amino aldehydes are outlined in Scheme 4. It is worth noticing that these reactions are not diastereoselective [5].

### 3.2. Amino ketones

**Scheme 5:**
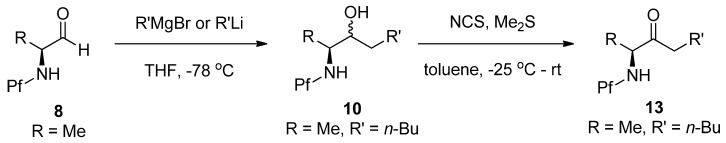
Ketone formation by nucleophilic addition to aldehyde [14].

*N*-Pf-Amino ketones can be prepared by nucleophilic addition either to a carboxylic ester [18], an aldehyde [14], an oxazolidinone [19] or an isoxazolidide [10]. However, the most widely used routes, presumably because of their good yields compared to others, are via an aldehyde or an oxazolidinone. A secondary alcohol is formed in the addition to an aldehyde and it needs to be further oxidized (Scheme 5).

**Scheme 6:**
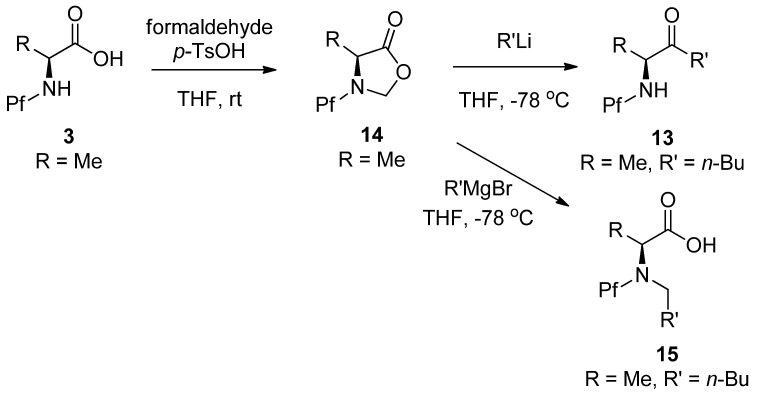
A method for preparation of *N*-Pf amino ketones through an oxazolidinone intermediate.

The advantage of proceeding through the oxazolidinone is the temporary full protection of nitrogen. The ketone can be formed by treatment with an alkyl lithium, for example. The corresponding Grignard reaction leads to *N*-alkylated product, presumably through an iminium ion intermediate [19](Scheme 6).

## 4. Enolization and Alkylation

**Figure 3:**
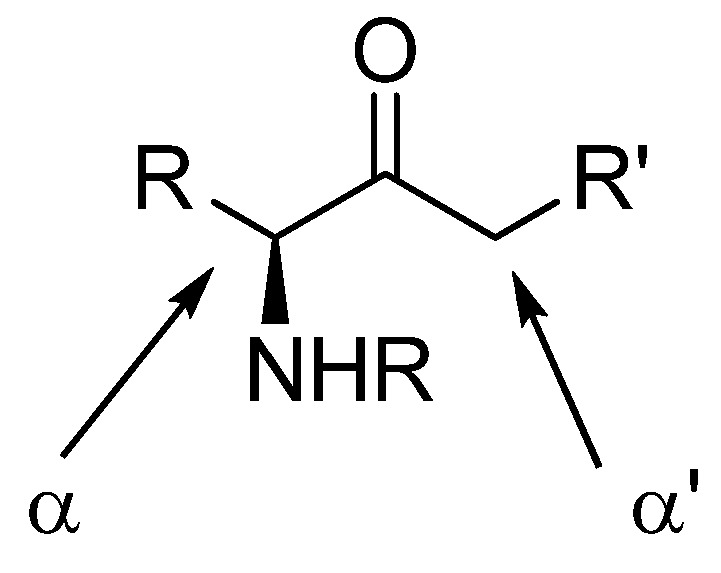
Easily enolizable positions in amino ketones.

Enolization and alkylation are important reactions in synthesis, especially if they can be performed regioselectively. α-Amino ketones possess two enolizable positions (Figure 3), often leading to poor selectivity in their alkylations. Amide- and carbamate protected aminoketones are easily alkylated at the α-carbon, which is often undesired. Their regioselective enolization in the α’-carbon is possible by kinetic control using LDA as the base.

Pf directs the regioselectivity of enolization and alkylation in both cyclic and acyclic compounds. The Pf protected amino ketones are deprotonated and alkylated solely at α’-carbon. This feature of the Pf group has been most widely utilized. Enolization is commonly performed with KHMDS as a base and alkyl halides as electrophiles [14].

### 4.1. Stereoselectivity of alkylation

**Scheme 7:**
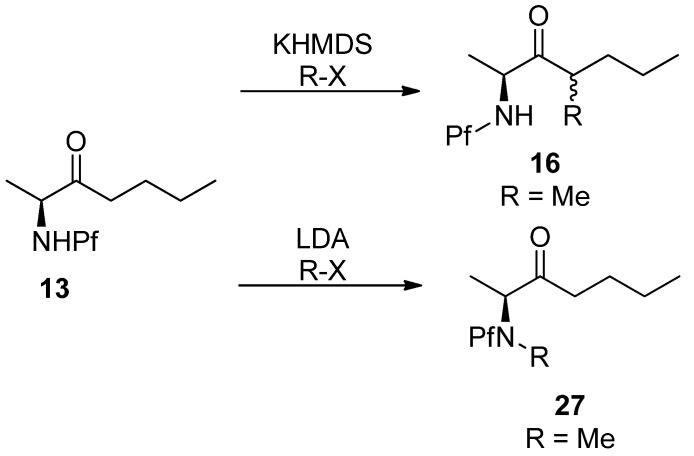
Regioselectivity of amino ketones is due to the base.

In general, the diastereoselectivity in alkylations of *N*-Pf-amino compounds is poor. The main product in the alkylation in Scheme 7 is *syn*, with up to 5:1 selectivity. It should be noted that the diastereoselectivity is dependent on the base, enolate cation and electrophile [14]. In spite of its size, the Pf group does not contribute to the selectivity.

**Scheme 8:**
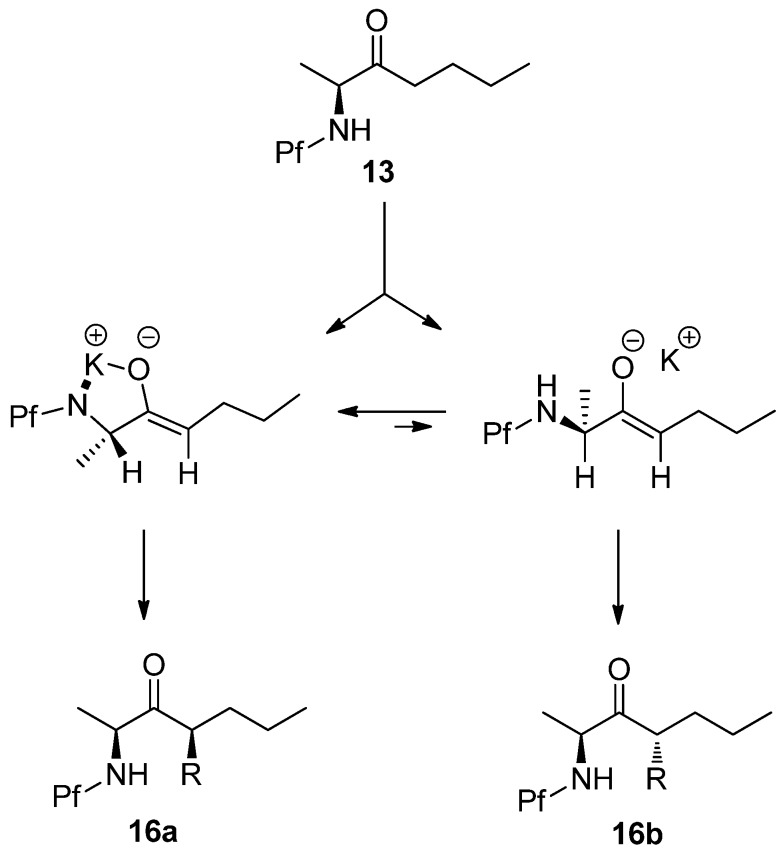
Chelation model [14].

The *syn*-selectivity of this alkylation can be explained by two transition state models. In the chelation model the nitrogen atom and the enolate oxygen atom are coplanar because of the electrostatic interaction with the K^+^-cation. In the other model the amino and methyl groups are *gauche* to the enolate oxygen. The electrophile approaches from the less hindered face, which forms the *syn* isomer in the chelation model and *anti* isomer in the non-chelated model. The chelated model is assumed to be more stable, because the steric interactions (allylic A^1,3^-strain) between the α- and vinyl protons and methyl and oxygen are small. (Scheme 8)

The influence of electrophiles in diastereoselectivity is presented in Table 1.

**Table 1:** Diastereoselectivity in regioselective alkylation.

Alkyl halide	Time / h	Yield^a^ / %	dr 16 (a:b)
MeI	3	94	2.2:1
BnI	6	80	5:1
Allyl bromide	6	77	5:1
Methyl α-bromopropanoate	8,5	38	2.2:1

^a ^**16a** + **16b**

### 4.2. Alkylation of nitrogen and synthesis of pipecolates

**Scheme 9:**
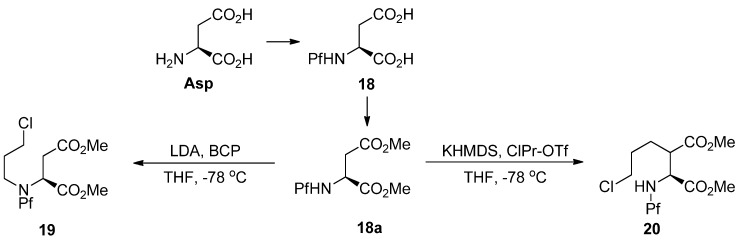
*N*- and *C*-alkylation of L-aspartate methyl ester.

The alkylation of nitrogen is often unwanted, but it can be utilized in preparing pipecolates from *N*-Pf-aspartate (Scheme 9) [3,20]. However, the electrophile must be particularly reactive, such as 3-bromochloropropane (BCP) [21], for *N*-alkylation to succeed. 

**Scheme 10:**
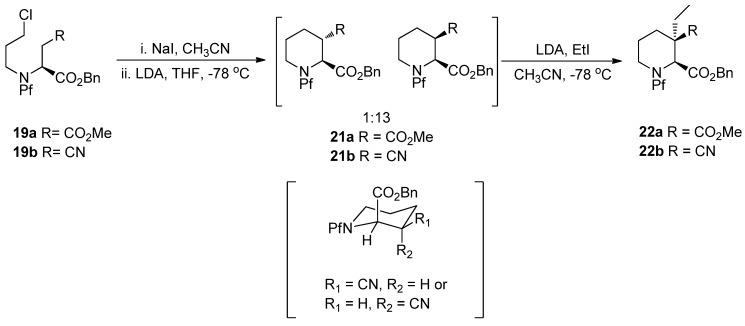
Alkylation of pipecolates [3].

The pipecolate structure formed in the ring forming reaction is rigid, so it can be alkylated diastereoselectively. The axial benzyl ester shields the upper side of the piperidine ring, which makes the electrophile approach from the lower side (Scheme 10).

### 4.3. Aids in alkylation

**Scheme 11:**
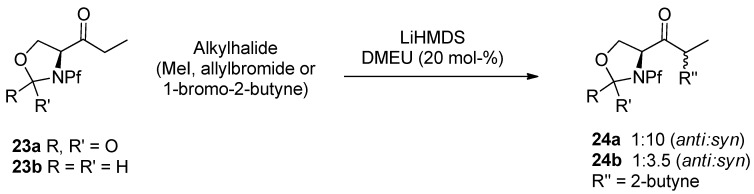
Alkylation of oxazolidinone and oxazolidide.

In some cases the alkylation may need a cosolvent, like DMPU, DMEU or HMPA, to break the enolate complex. For example the alkylation of serine derived oxazolidinone and oxazolidide by lithium and potassium enolates in THF has been helped to go forward with DMEU (Scheme 11). KHMDS yields both mono- and di-alkylated product. LiHMDS gives mainly monoalkylated product [10].

## 5. Alkylation of L-Aspartate and L-Glutamate

Enolization and alkylation of L-aspartate at the β-carbon is a robust and a straightforward reaction [22]. In this particular case, the Pf group has been reported to protect both the α- and β-centers from racemization. Diastereoselectivity of the alkylation is not good and cannot be explained unambiguously. An almost 1:1 mixture is generally formed, but excellent selectivities can also be achieved. For dialkylation, the Pf protecting group has recently been used as a directing group in the synthesis of polytheonamide B [23].

### 5.1. Stereoselectivity of alkylation of L-aspartate

Improving the stereoselectivity of alkylation of *N*-Pf-aspartate ester enolates has been attempted by fully protecting the nitrogen. The highest stereoselectivity (50:1 *syn:anti*/*anti:syn*) has been achieved by diprotecting the nitrogen with Pf and Bn [24]. In that case the selectivity is determined so that KHMDS gives dominantly the *anti*- while LiHMDS gives the *syn*-product. The impact. of the electrophiles on selectivity are presented in Table 2.

**Table 2:** Alkylation of *N*-Pf-Bn-D-aspartate.

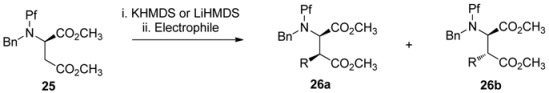			
Electrophile	KHMDS, -23 °C	LiHMDS, -23 °C	LiHMDS, -78 °C
	26 (a:b)	26 (a:b)	26 (a:b)
Allyl iodide	1:10	23:1	-
MeI	2:1	>50:1	>50:1
BnBr	>1:50	>50:1	>50:1

**Figure 4:**
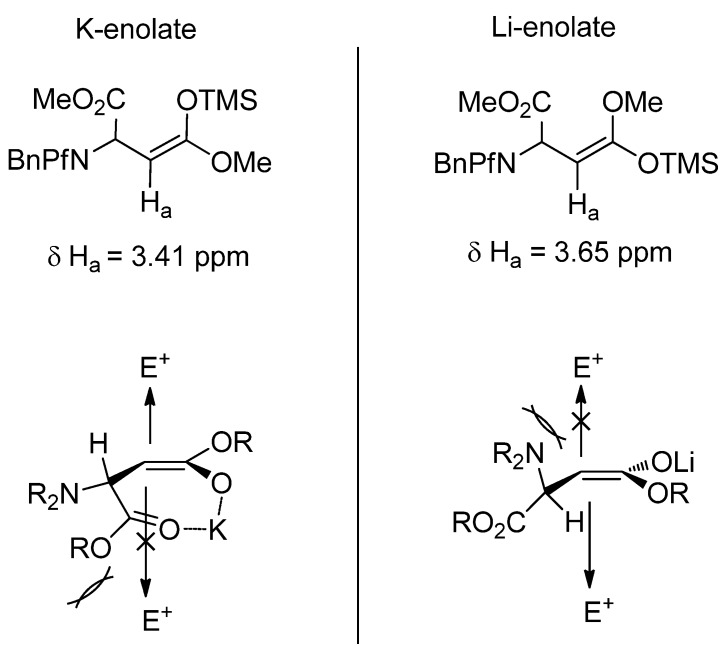
Silyl ketene acetals of potassium and lithium enolates, and the geometries that affect the stereoselectivity of alkylation.

It has been shown by trapping the enolate formed in the reaction with TMSCl that potassium and lithium bases form different enolates. Potassium base forms a *Z*-enolate and a cyclic chelated transition state. The *anti* product arises when the electrophile approaches from the less hindered face opposite to the Pf group. Lithium enolate has a *E*-geometry so it can not chelate. The electrophile approaches from the less hindered face and the product is *syn* (Figure 4) [24]. 

### 5.2. Hydroxylation and amination of L-aspartate

Hydroxylation (Table 3) and amination of N-Pf-aspartate enolates have shown that stereoselectivity is dependent on interactions of many factors. The stereogenic center is affected by the enolate geometry, counter cation, reaction conditions, cosolvent, electrophile and the ionization degree of the amino group [25].

**Table 3:** Hydroxylation of L-aspartate methyl ester.

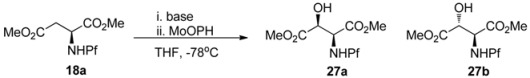				
18a	Base (mol %)	Cosolvent	27 (a*:*b)	Yield 27 (%)^a^
1	KHMDS (180)	-	3:1	90
2	LHMDS (300)	-	1:8	65
3	LDA (300)	-	2:1	45
4	LTMP (180)	-	1:2	22
5	LHMDS (300)	DMPU	8:1	92
6	LHMDS (300)	HMPA	11:1	74
7	LHMDS (300)	DME	1:2.5	70
8	LHMDS (300)	TMEDA	1:8	80
9	LHMDS (300)	PMDET	1:5	75
10	KHMDS (180)	18-crown-6	-	
11	LHMDS (300)	12-crown-4	-	
12	n-BuLi (100) / LHMDS (300)	-	1:20	60
13	n-BuLi (100) / LHMDS (300)	HMPA	2:1	95

^a ^Yield (%) mixture of epimers

The use of Davis’ reagent as the hydroxylating agent gives a 1:1 mixture of diastereomers under all conditions examined. *N*-Pf-Aspartate *tert*-butyl ester and free acid react with poor selectivity. Enolates generated with LiHMDS do not react at all with MoOPH [25]. 

**Figure 5:**
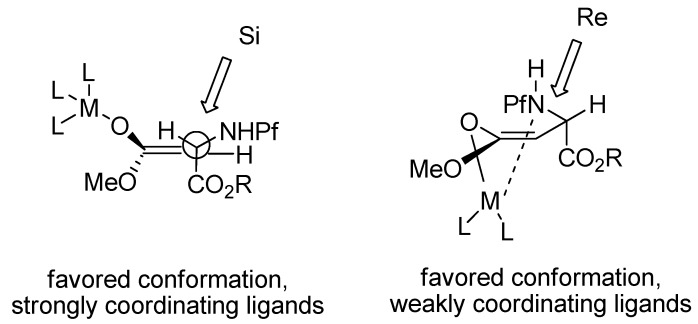
Transition state models for enolates.

The selectivity has been explained by mechanistic studies. The enolate is presumably in equilibrium with an open and a chelated form, in which the Pf group is in an equatorial position. Cosolvents like DMPU and HMPA that coordinate strongly to metals prefer the open form, as well as K^+^ as the countercation. Poorly coordinating ligands like HMDS, THF or *n*-BuLi as the base, prefer the chelated form. Hard, neutral cosolvents such as HMPA and DMPU form stronger complexes with Li^+^ than neutral softer bases such as DME, TMEDA or PMDET [25] (Figure 5). An electrophile such as MoOPH that does not complex with the enolate metal cation, approaches from the less hindered face. Ligands that make too strong complexes with the counter cation of the enolate form a naked enolate that does not react.

The stereoselectivity of amination is poor, at least when DTBAD or DBAB are used. With these reagents in the conditions described in Table 3, a 1:1 mixture of diastereomers is formed in nearly every case. However, the diastereomers can be easily separated by crystallization, and epimerized to the other isomer. Entry 6 (Table 3) with DTBAD as the electrophile is an exception, where diastereoselectivity is as high as 30:1 (*anti*:*syn*). In all cases the major isomer in amination is *anti* [25].

**Scheme 12:**
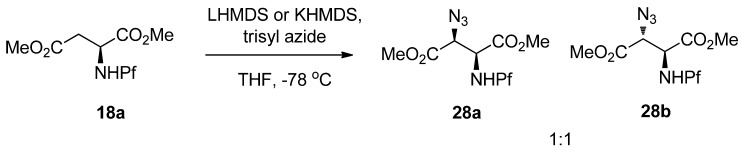
Amination of *N*-Pf-aspartate dimethyl ester.

### 5.3. Substituted prolines from L-glutamate

**Scheme 13:**
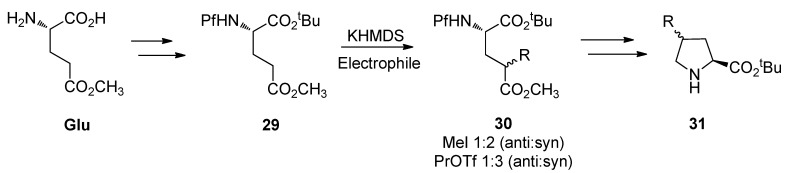
Synthesis of 4-substituted prolines.

The regioselective enolization and alkylation of L-glutamic acid have been used in enantiospecific synthesis of 4-substituted prolines (Scheme 13). The *anti*:*syn* stereoselectivity in this alkylation varies from 1:2 to 1:3 [26].

The stereo center in γ-methyl glutamate **30** can be epimerized to increase the proportion of the major *syn* isomer, but the diastereomeric ratio of the *anti* and *syn* isomers could not be inverted. The effect of base in the epimerization is presented in Table 4.

**Table 4:** Epimerization of γ-methyl glutamate.

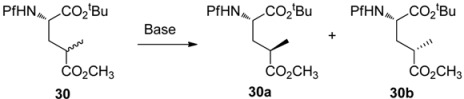			
Base	Temperature / °C	Time / h	30 (a:b)
KHMDS	-78	0.7	1:3
LDA	-78	0.7	1:2.3
BMDA^a^	-78	2	1:6.5
NaOMe	20	20	1:2.7

^a^ BMDA = bromomagnesium diisopropylamide

#### 5.3.1. Alkylation of hydroxyproline

**Scheme 14:**
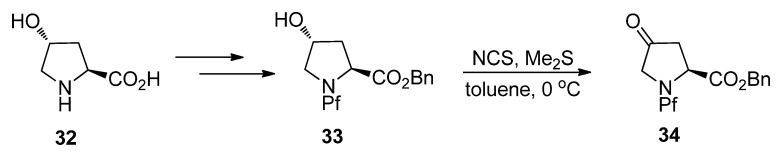
Oxidation of hydroxyproline **32**.

*N*-Pf-Hydroxyproline can be oxidized to corresponding ketone (Scheme 14), that can be further regioselectively enolized and alkylated. The stereoselectivity is again due to both the base and the electrophile [27] and the effects of bases and electrophiles on the stereoselectivity of the alkylation are presented in Table 5.

**Table 5:** Alkylation of *N*-Pf-proline ketone and its stereoselectivity [27].

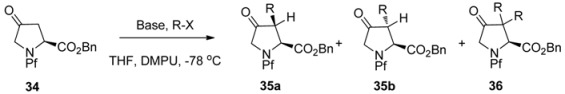				
34	Base (Mol %)	RX (mol %)	Yield 35 (%) (b:a)	Yield 36 (%)
1	NaHMDS (140)	MeI (500)	65 (3:1)	14
2	KHMDS (200)	MeO_2_CCH_2_Br (220)	81 (1:2)	-
3	NaHMDS (140)	H_2_C=CHCH_2_I (500)	54 (>6:1)	5
4	KHMDS (280)	PhCH_2_Br (300)	24 (1:1)	2
5	KHMDS (400)	MeI (1000)	-	82
6	KHMDS (400)	EtO_2_CCH_2_I (1000)	-	52
7	KHMDS (400)	H_2_C=CHCH_2_I (1000)	-	69
8	KHMDS (400)	PhCH_2_Br (670)	-	29

**Scheme 15:**
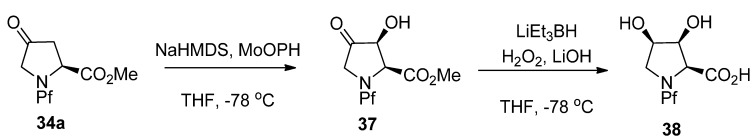
Synthesis of *N*-Pf-dihydroxyproline.

N-Pf-Proline ketone **34a** can be hydroxylated regio- and stereoselectively into alcohol **37** and finally into all *cis*-dihydroxyproline **38** [28] (Scheme 15).

The above-presented regioselective enolization of *N*-Pf-proline ketone can also be utilized in the synthesis of 4-substituted-3,4-didehydroprolines [29]. Here the ketone is transformed into the corresponding enol triflate, which is then coupled to a suitable substituent (Table 6).

Similar enolisation protocols have been utilised in the synthesis of kainic acid derivatives [30,31].

**Table 6:** Synthesis of 4-substituted-3,4-didehydroprolines.

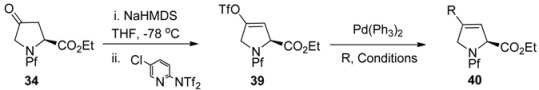				
39	R		Conditions	Yield 40 (%)
1	CO_2_H	CO/KOAc	DMF, rt, 66 h	87
2	CO_2_Me	CO/MeOH	DMF, reflux, 2 h	81
3	Ph	PhSnMe_3_	THF, reflux, 16 h	60
4	Me	SnMe_4_	THF, reflux, 48 h	5-50
5	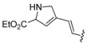	Bu_3_SnCH=CHSnBu_3_	THF, reflux, 48 h	40

## 6. Reduction and Oxidation of *N*-Pf-amino Compounds

Ketones, triple bonds and esters present in *N*-Pf-amino compounds can be reduced to alcohols, alkenes and aldehydes without removing the Pf protection. However, it should be remembered that the Pf group is readily hydrogenolyzed in Pd/C-hydrogenation and it is not stable under acidic ester hydrolysis. It is often possible to perform the hydrogenolysis of the Pf group and protection with Boc in the same step, effectively changing the protecting group to Boc in one operation.

### 6.1. Hydride reductions

In the hydride reduction of the carbonyl in *N*-Pf-dimethyl proline ketone, the stereochemistry of the product is due to reducing agent. LiAlH_4_ and NaBH_4_ lead to different products, although with poor diastereoselectivity [27].

**Scheme 16:**
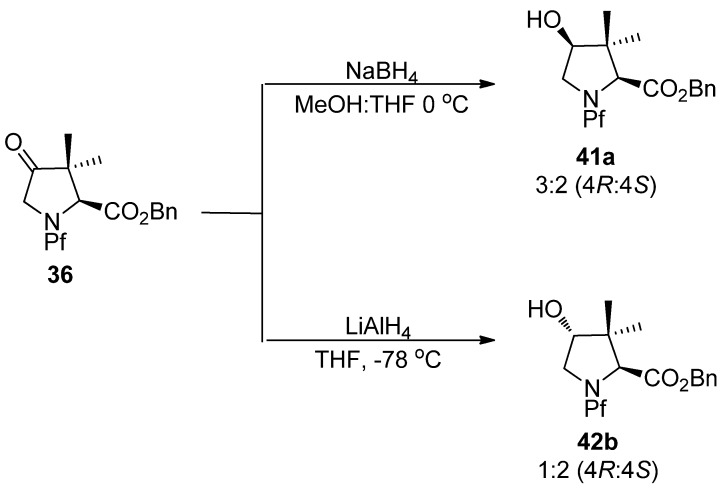
Selectivity in the reduction of substituted prolines.

**Scheme 17:**
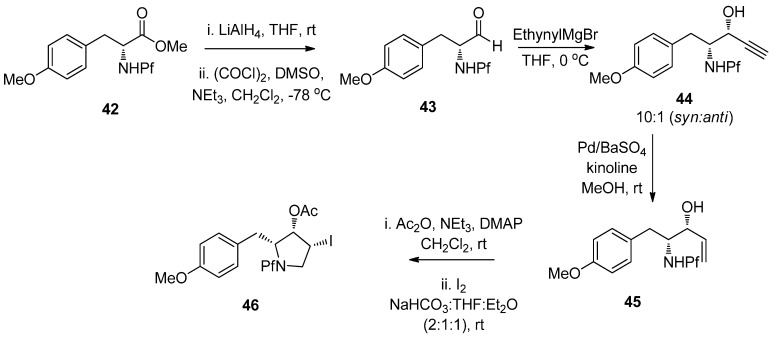
Synthesis of (+)-anisomycin.

*N*-Pf-D-Tyrosine was used in the synthesis of (+)-anisomycin [32]. The pyrrolidine structure in the target molecule was formed by using both hydride and palladium-catalyzed reductions in addition to Swern oxidation. No epimerization was observed and the Pf stayed intact (Scheme 17). 

**Figure 6:**
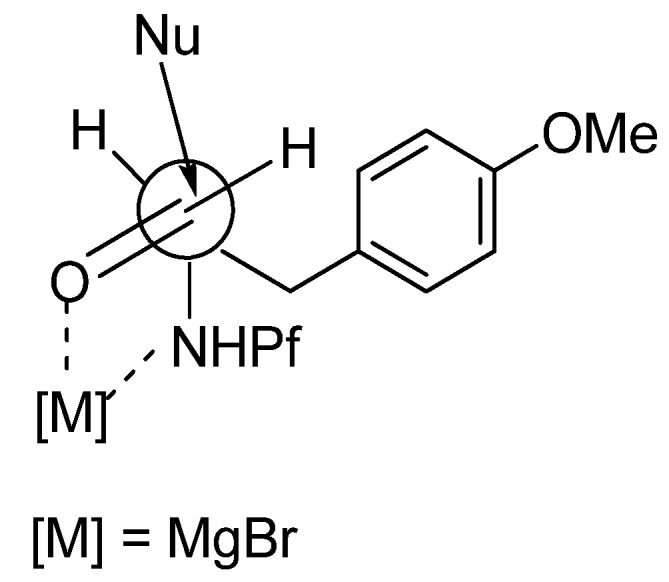
The transition state of *syn*-selectivity in Grignard-reaction.

The main product in the Grignard reaction is *syn*, which can be explained by chelation controlled Cram-model (Figure 6). The steric bulk of the Pf group might be the cause of *syn*-selectivity (*syn*:*anti* 10:1). In the transition state the nucleophile attacks from the less hindered face opposite to the Pf amine.

**Scheme 18:**
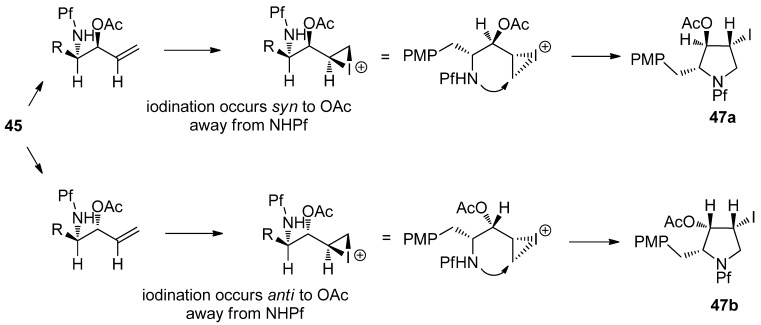
Proposed mechanism for the stereoselective iodoamination.

The pyrrolidine ring is formed from the alcohol derived acetates by iodination of the double bond in compound **45**. The stereoselectivity of the iodination is without a doubt due to the steric hindrance posed by the Pf group. An epi-iodinium ring is formed on the less hindered side of the double bond regardless of the spatial arrangement of the acetate (Scheme 18).

Anisomycins have been made by a related reaction sequence, in which the same allyl alcohol intermediate is utilized [32]. In the previous synthesis the stereochemistry of the product (*syn*) was determined in the addition step to aldehyde **9**, but in this case it can be directed to form either *anti* or *syn*. The addition is carried out by Gringard reaction with vinyl magnesium bromide, in which a diastereomeric mixture of alcohols **45** is formed. The alcohol mixture is oxidized to allylic ketone **48** by Swern oxidation. The formed ketone can be reduced stereoselectively. Results concerning the relation between the conditions and the stereochemistry of the product are presented in Table 7.

As can be seen, the best stereo- and regioselectivity is attained with (*S*)-BINAL that favours the *anti*-product. By Felkin-Ahn-model predicted *syn*-product is formed with BH_3_^.^(CH_3_)_2_S. According to the polar Felkin-Ahn-model the most electron withdrawing group is placed where the largest substituent is according to classical FA-model.

**Table 7:** Effect of the conditions and reagents in the stereoselectivity of the reduction.

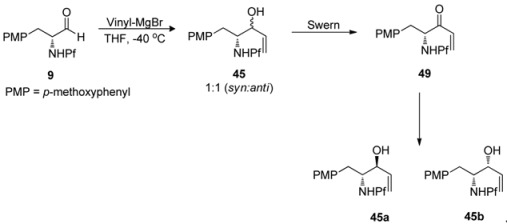				
49	Reagent	Conditions	45 (a:b)	Yield 45 (%)
1	BH_3_-(CH_3_)_2_S	Toluene, -78 °C	1:9	91
2	(*S*)-BINAL	THF, -78 °C	>95:1	89
3	(*R*)-BINAL	THF, -78 °C	1:6	82
4	NaBH_4_	THF, -10 °C		*a*
5	DIBAL-H	THF, -78 °C		*a*
6	L-Selectride	THF, -78 °C	1:5	45
7	KS-Selectride	THF, -78 °C	1:6	38
8	Red-Al	THF, -40 °C	1:8	20

*a*: product that arises from 1,4-addition

**Figure 7:**
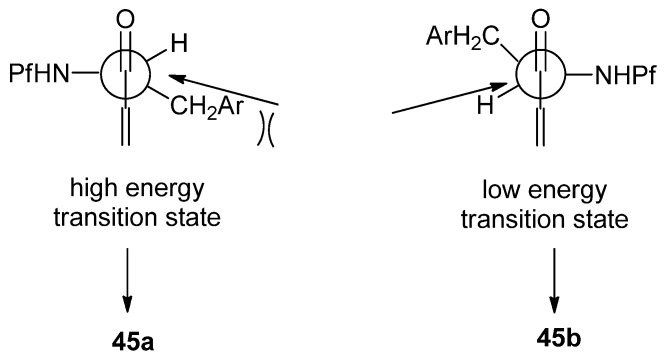
Comparison of Felkin-Ahn model in selective reduction.

The main product is formed when the nucleophile approaches the C-H bond in a Bürgi-Dunitz angle from the less hindered side. This decreases the energy of the transition state (Figure 7). Simple hydride reducing agents such as NaBH_4_ and DIBAL-H favor 1,4-reduction instead of 1,2-reduction [32]. 

### 6.2. Reductive amination

Reductive amination was the key step in the synthesis of indolizidinone amino acids [33]. The Pf group was hydrogenolyzed from the Claisen condensation product **50**, originally formed by regioselective enolization of *N*-Pf-glutamic acid **29**. The hydrogenolysis was followed by ring closure to **51** and further cyclization to lactams **52a,b**. The diastereoselectivity of the reductive amination is due to the pressure of hydrogen gas and the acid used (Table 8).

**Table 8:** The effect of hydrogen pressure and the acid on the diastereomeric ration in reductive amination.

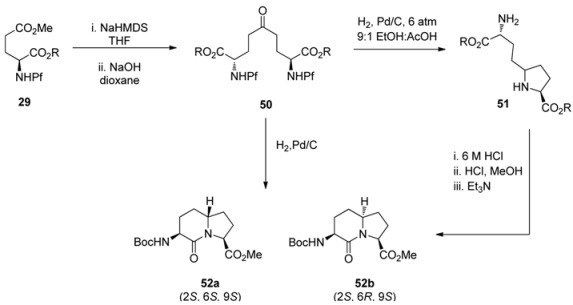					
51	H_2_ pressure/atm	Acid	Yield 52a (%)	Yield 52b (%)	dr (46a:46b)
1	1	TFA	41	22	65:35
2	6	TFA	52	10	84:16
3	11	TFA	54	6	90:10
4	1	6 M HCl	45	22	67:33
5	6	6 M HCl	64	8	89:11
6	11	6 M HCl	66	3	96:4

### 6.3. Dihydroxylation

In general, dihydroxylation of allylic amines is not stereoselective, on which the Pf group has no impact. The dihydroxylation of *N*-Pf-amino alkene **53** with KMnO_4_ or OsO_4_ forms a mixture of diastereomers [4].

**Scheme 19:**
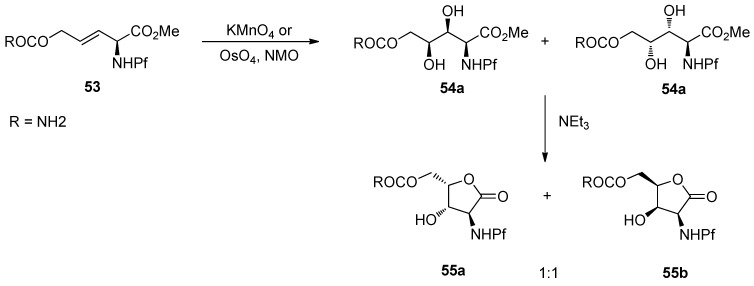
Dihydroxylation of *N*-Pf-amino alkene.

With the Sharpless DHQ- and DHQD-ligands the diastereoselectivity is improved only to 2:1 and 1:2.6 (**55a:b**). If a similar alkene **56** is subjected to conjugate addition, a mixture of different products is formed dependent on the temperature and other conditions. Considering the structures of the products, one can note that C-C-bond forming reaction is slow and the Pf protected nitrogen is reactive towards intramolecular electrophiles [4].

**Scheme 20:**
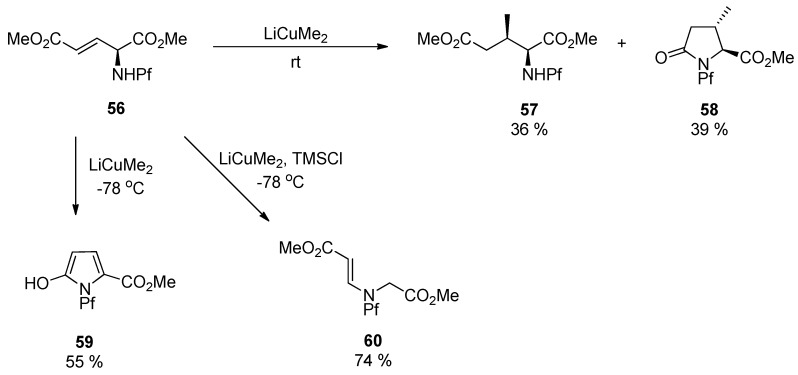
Conjugate addition and the products.

## 7. Pf in Ring Forming Reactions

### 7.1. Intramolecular ring formation

**Scheme 21:**
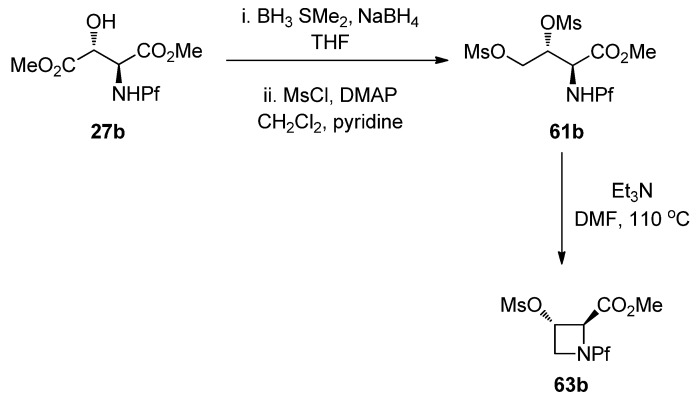
Ring formation from *anti*-*N*-Pf-hydroxyaspartate methyl ester.

L-*N*-Pf-Hydroxy asparagine methyl ester can be used, for example, in the synthesis of aziridine structure that is present in bioactive mitomycins [34]. Depending on the conditions an azetidine is formed as a side product (Table 9). Anti *N*-Pf-Hydroxy aspartate methyl ester is cyclized to a four-membered ring only and not at all to aziridine (Scheme 21).

**Table 9:** Intramolecular cyclization of *N*-Pf-aspartate to aziridine or azetidine [34].

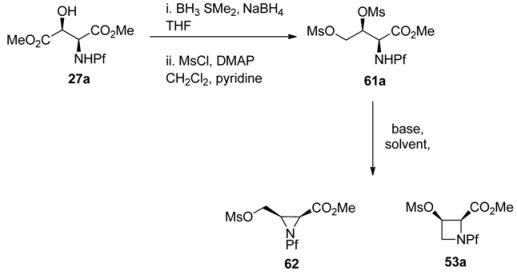			
61a	Base, solvent	T / °C	62:63
1	Et_3_N, THF	Reflux	No reaction
2	Et_3_N, DMF	80	4:1
3	Et_3_N-LiClO_4_, DMF	92	7:1
4	Et_3_N-LiClO_4_, THF	Reflux	25:1
5	LiClO_4_, s-collidine, dioxane	75	100:0

**Figure 8:**
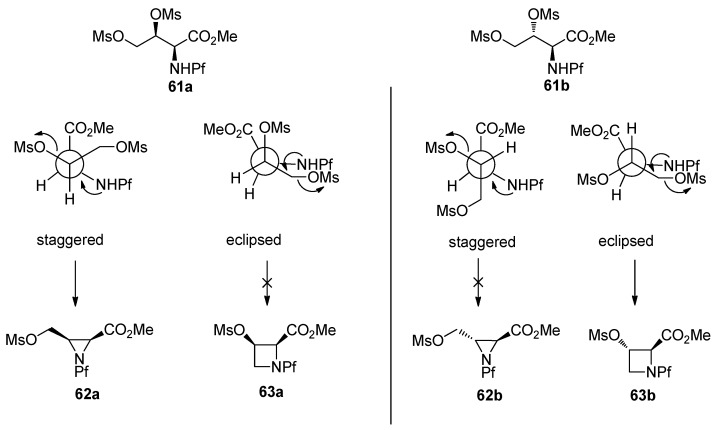
Newman-projections that explain the differences in stereoselectivity.

The stereochemistry of the starting material has an effect on the outcome of the reaction. That can be explained by Newman projections that illustrate the contribution of the Pf groups size to the more favored conformation (Figure 8). The cyclization of dimesylate **61a** to aziridine proceeds presumably through a staggered conformation since the eclipsed conformation has two unfavorable interactions, namely CO_2_Me-OMs and NHPf-CH_2_OMs. In the case of dimesylate **61b**, the cyclization is forced to proceed through the eclipsed conformation, since there are unfavorable interactions in the staggered conformation, namely Pf-CH_2_OMs or Pf-carboxymethyl.

Pf can be removed by hydrogenation (H_2_, 50 psi, Pd/C) without opening the ring structure. Removal of protection from Bn-protected aziridine leads normally to ring opening.

### 7.2. Formation of indanones by metal-halogen-exchange

**Scheme 22:**
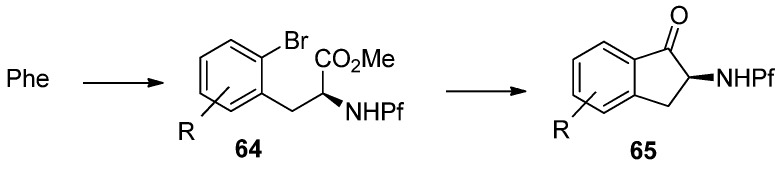
Synthesis of indanones from N-Pf-bromophenylalanine derivatives.

Cyclization of *N*-Pf-bromophenylalanine is the key step in the synthesis of indanones (Scheme 22) [35].

**Scheme 23:**
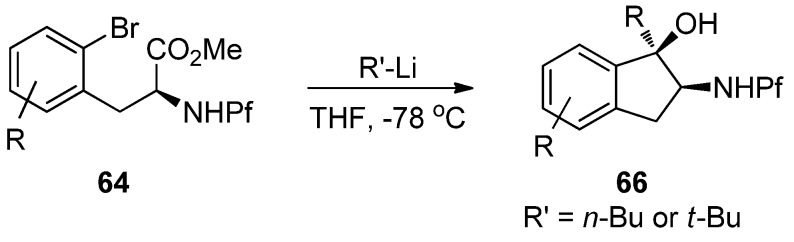
Treating starting material with excess BuLi.

The reaction is based on metal-halogen-exchange, in which the electrophilic halogen is displaced with a nucleophilic metal. However, treating the starting material with excess n-BuLi does not lead to the desired product, but to indanol and starting material (Scheme 23).

One problem in the reaction is the free proton on nitrogen. The product is not formed at all if a stoichiometric amount of *n*-BuLi is used. On the other hand, a mixture of different products is formed if a free acid is used.

**Scheme 24:**
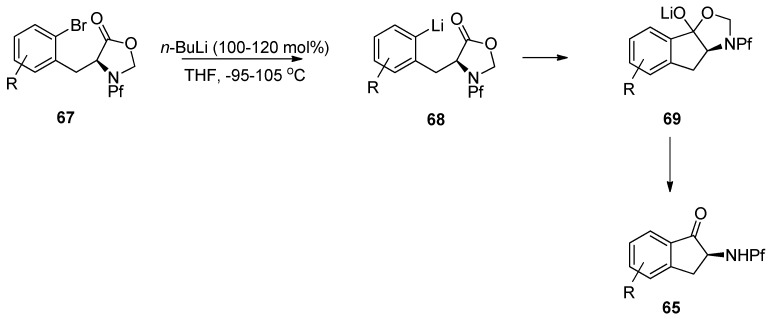
The successful synthesis of indanones through intramolecular cyclization of N-Pf-bromophenylalanine.

According to these observations it can be concluded, that deprotonation of *N*-Pf-amine occurs faster than metal-halogen exchange or nucleophilic addition to ester. The desired product is achieved by protecting the amine as oxazolidinone (Scheme 24) [35].

### 7.3. Cyclic sulfamidates as protection for amines

Alkylation of the amine present in derivatives of α-amino compounds and intramolecular cyclizations are often unwanted side reactions. The nucleophilicity of the nitrogen atom in *N*-Pf compounds can be reduced by fully protecting it. This protection can be temporary when it is necessary for a single reaction step to succeed, but it can also be used in the whole path and removed at the end of the sequence. 

**Figure 9:**
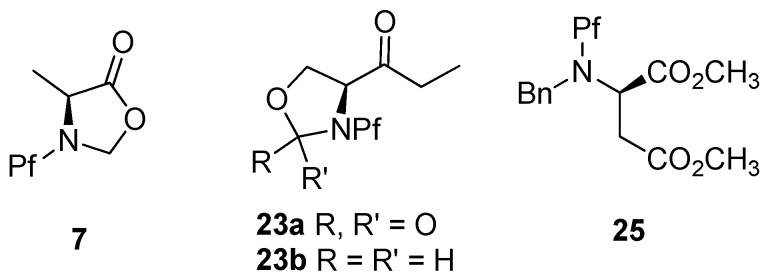
The most common ways of double protecting the nitrogen present in *N*-Pf-amino compounds.

Derivatives of serine are often used as oxazolidinones **23a** or oxazolidines **23b** because of its hydroxyl group [10,15], which protects the nitrogen as well. In some cases the hydroxyl is protected as benzyl ether [9]. Asparagine acids have been diprotected with Pf and benzyl, which has even proven to improve the stereoselectivity of the alkylation. Alanine is often protected as isoxazolidinone **7** (Figure 9) [36].

**Figure 10:**
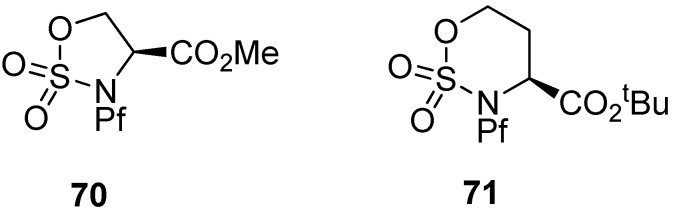
Cyclic sulfamidates derived from *N*-Pf-serine and *N*-Pf-homoserine.

Cyclic sulfamidates have been made from *N*-Pf-serine and *N*-Pf-homoserine (Figure 10), and they have been used in the preparation of substituted amino acids [37,38,39].

**Scheme 25:**
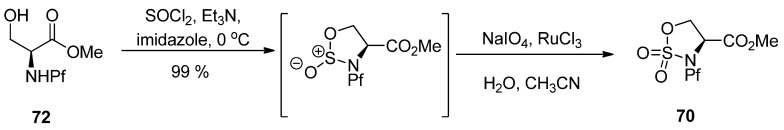
Formation of N-Pf-serine derived sulfamidate.

Sulfamidate is formed from the free alcohol of serine or homoserine methyl ester by thionyl chloride and metal catalyzed oxidation (Scheme 25) [37].

Sulfamidates are electrophiles and can be reacted with different nucleophiles. Ring opening by β-ketoester leads to racemic γ-acyl amino acid **72** in addition to side product **73** (Table 10).

**Table 10:** Addition of β-ketoester to sulfamidate **70** [37].

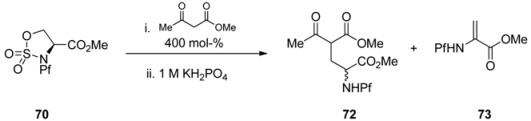			
70	Conditions	Yield 72 (%)	Yield 73 (%)
1	K_2_CO_3_, DMF, rt, 3 d	-	100
2	K_2_CO_3_, THF, rt, 3 d	30	-
3	Cs_2_CO_3_, THF, rt, 5 d		100
4	K_3_PO_4_, DME, rt, 18 h	43	-
5	NaH, THF, 60 °C, 6 h	51	-
6	NaH, DME, 60 °C, 4 h	67	

**Scheme 26:**
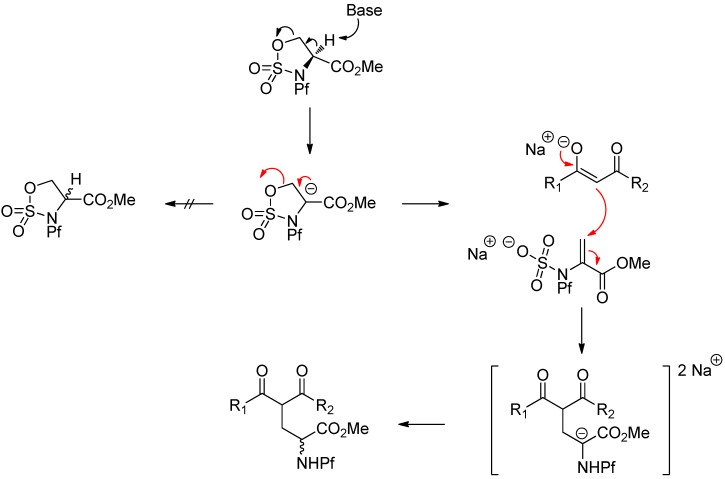
Proposed mechanism for the ring opening that leads to racemization.

A proposed mechanism for the racemization is shown in Scheme 26.

**Scheme 27:**
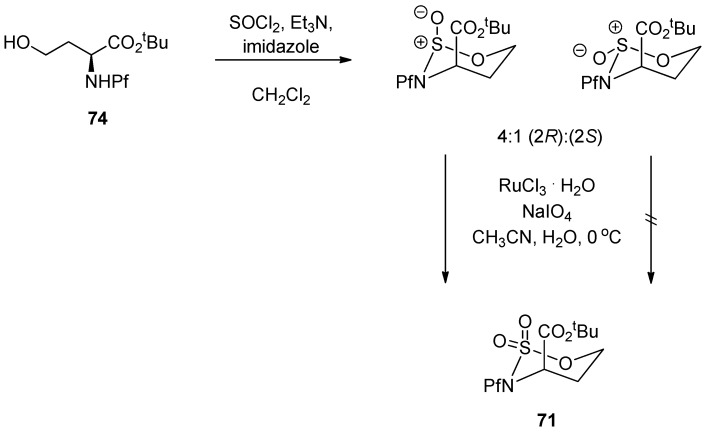
Preparation of *N*-Pf-homoserine *tert*-butyl ester sulfamidate and its conformation.

Homoserine derived *N*-Pf-sulfamidate has been observed to be in chair conformation, typical also with similar cyclic sulfamidates [40]. The CO_2_R group is axial as in the corresponding pipecolates [21] (Scheme 27).

**Table 11:** Conditions affecting the nucleophilic addition to sulfamidate [37].

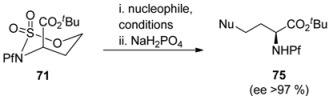			
71	Nucleophile	Conditions	Yield 75 (%)
1	NaN_3_	DMF, 60 °C, 24 h	83
2	imidazole	NaH, DMF, 60 °C, 24 h	50
3	imidazole	DMF, 60 °C, 24 h	56
4	imidazole	CH_3_CN, 75 °C, 30 h	65
5	morpholine	NaH, DMF, 60 °C, 24 h	85
6	morpholine	CH_3_CN, 75 °C, 30 h	95
7	piperidine	DMF, 60 °C, 24 h	80
8	piperidine	CH_3_CN, 75 °C, 30 h	90
9	PhNH_2_	CH_3_CN, 75 °C, 30 h	85
12	PhSH	NaH, DMF, 60 °C, 24 h	56
13	PhSH	CH_3_CN, 75 °C, 30 h	0

**Figure 11:**
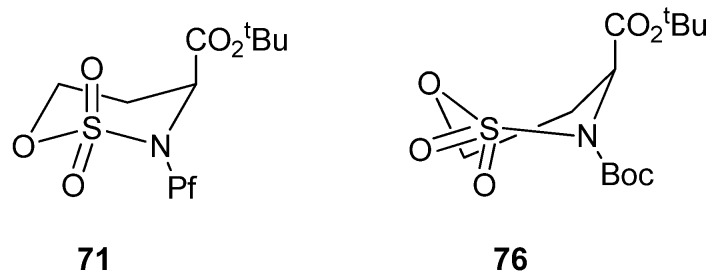
Conformations of *N*-Pf sulfamidate and *N*-Boc sulfamidate.

Boc-protected sulfamidate **76** is more reactive towards nucleophiles than the corresponding Pf-protected sulfamidate **71** [41]. This enhanced reactivity is thought to arise partly from the inductive electron withdrawing effect of the N-substituent and partly from the different conformations that *N*-Boc and *N*-Pf compounds adopt. The *N*-Boc compound **76** has a preference for twisted boat, whereas the *N*-Pf derivative **71** favors chair comformation (Figure 11) [41].

**Table 12:** Differences in reactivities of *N*-Pf and *N*-Boc-sulfamidates in nucleophilic addition [38].

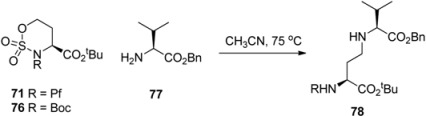				
Sulfamide	Solvent	Conversion (%)	Yield 78 (%)	Time/h
**76**	MeCN	99	95	3
**76**	CHCl_3_	99	60	5
**71**	MeCN	99	72	28
**71**	CHCl_3_	20	20	73

## 8. Metal Catalyzed Reactions and Couplings

There are some published examples where *N*-Pf-amino aldehydes have been used in metal-catalyzed couplings. Here are presented Horner-Wadsworth-Emmons and [3+3]-coupling to titanium homoenolates as examples.

### 8.1. Horner-Wadsworth-Emmons

**Scheme 28:**
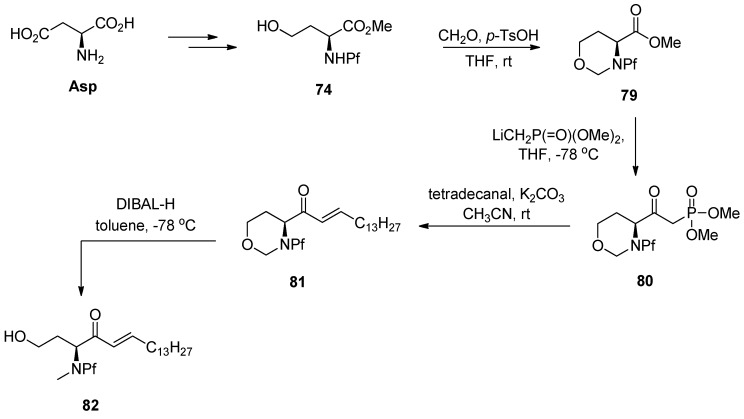
HWE.

Horner-Wadsworth-Emmons (HWE) is a coupling reaction that has been used in the synthesis of certain sphingosine derivatives using *N*-Boc-amino acid derived β-ketophosphonates [42]. It also works with the corresponding *N*-Pf-compounds [6] (Scheme 28).

**Figure 12:**
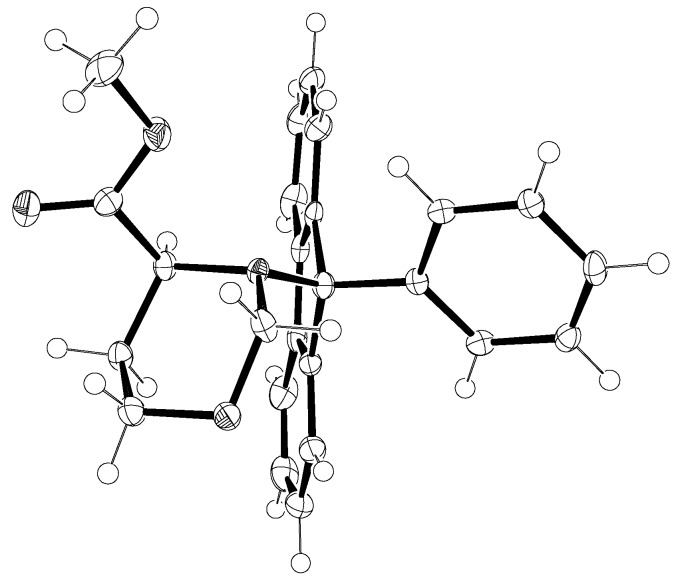
The crystal structure of compound **79**.

In this study certain crystal structures of *N*-Pf-compounds were determined. The crystal structure of compound **79** is presented in Figure 12. It can be observed that the phenyl group of Pf is almost perpendicular to the fluorene. The same phenomenon is true for both cyclic and acyclic compounds. During this study it became arguable that the axial position of the electronegative α-substituent in cyclic compounds might be due to the anomeric-like effect it experiences [6]. This would explain the equatorial position of the hydrogen more accurately than the original explanation [5].

### 8.2. [3+3]-Titanium homoenolate coupling

**Scheme 29:**
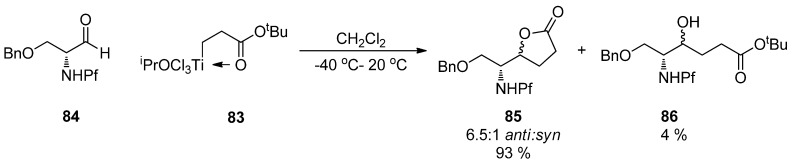
The coupling of *N*-Pf-*O*-Bn-serinal **84** and titanium homoenolate **83**.

The [3+3]-coupling of titanium homoenolates **83** to *N*-Pf-*O*-Bn-serinal **84** is an example of the less common additions to aldehydes (Scheme 29). The diastereoselectivity of the reaction is in accordance with the previously modeled *anti*-selectivity of the nucleophilic addition to *N*,*N*-dibenzylserinal [9].

### 8.3. N-Pf-Amino acids as ligands in the Zn-catalyzed addition to aldehydes

**Figure 13:**
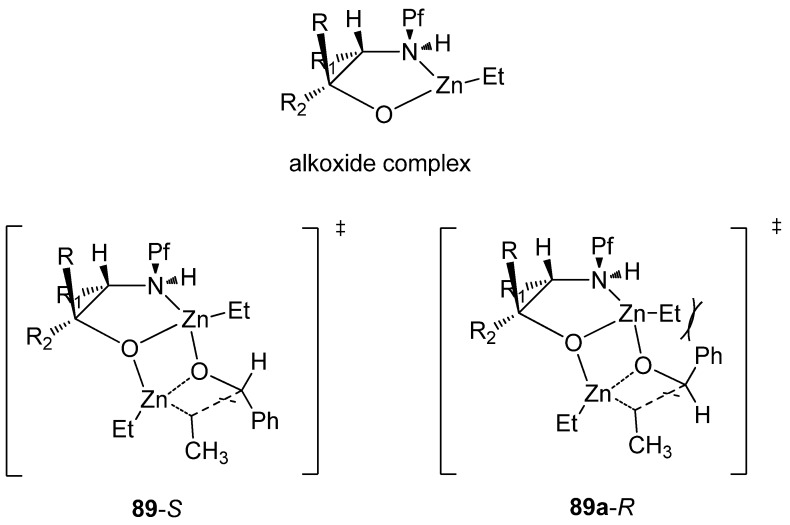
The assumed complexes.

It is very rare to use the Pf protected amino compounds as ligands in metal-catalyzed reactions. *N*-Pf-amino alcohols have been used as chiral ligands in secondary alcohol forming zinc-catalyzed additions to aldehydes (Table 13) [36]. A *syn* configuration in the ligand gives the *S*-product, while *anti* ligands give the *R*-product. The proposed mechanism for the catalytic cycle begins with the complexation of diethyl zinc and the ligand (Figure 13). The Pf group is supposed to act as a conformational lock for this amino alkoxide complex. This leads to the formation of a di-zinc complex, which undergoes intramolecular ethyl migration to give a catalyst-product –complex. Ferrocenyl, γ- and δ-amino alcohols and chiral piperazines have been used before in this reaction [36]. 

**Table 13:** *N*-Pf-Amino alcohol ligands in Et_2_Zn-catalyzed addition to benzaldehyde.

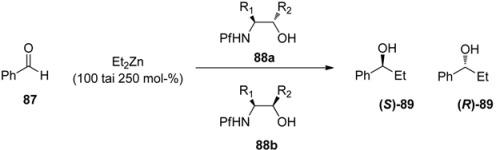					
Experiment	Ligand 88 (3 mol-%)	R_1_	R_2_	% ee	Configuration of the product
1	a	Me	Ph	76	*S*
2	b	Me	Ph	29	*R*
3	a	Me	Ferrocenyl	76	*S*
4	b	Me	Ferrocenyl	17	*R*
5	a	Me	Naphtyl	40	*S*
6	b	Me	Naphtyl	66	*S*
7	a	Me	t-Bu	97	*S*
8	b	Me	t-Bu	14	*S*
9	a	Me	Ph_3_C	90	*S*
10	b	Me	Ph_3_C	18	*S*

### 8.4. Selective hydrolysis of esters with zinc bromide

**Scheme 30:**
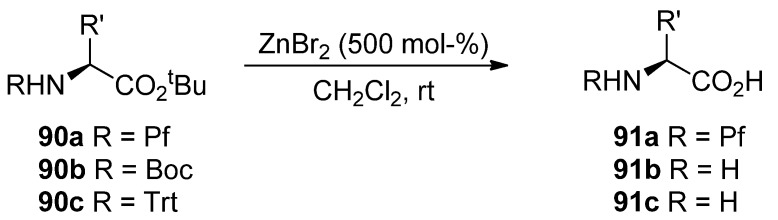
Hydrolysis of *tert*-butyl esters present in *N*-protected amino acids.

*tert*-Butyl esters can be selectively hydrolyzed to free acids with ZnBr_2 _in the presence of a Pf protected amine [43]. *N*-Boc and *N*-trityl amino compounds hydrolyze to free amines in the same conditions in contrast to *N*-Pf-compounds (Scheme 30).

**Scheme 31:**
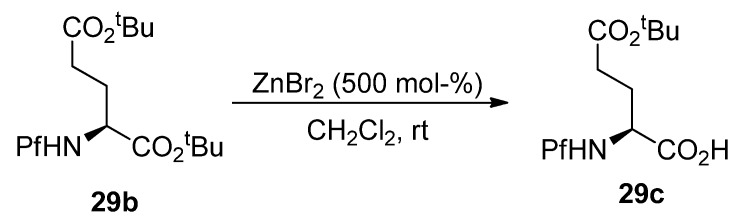
Selective hydrolysis of α-*tert*-butyl ester.

*tert*-Butyl ester in the α-position of compound **29b** can be selectively hydrolyzed with ZnBr_2_ (Scheme 31).

## 9. Phenylfluorenyl in Total Synthesis

Many enantiospecific syntheses have been possible thanks to the regioselective enolization and alkylation of *N*-Pf-amino compounds. This reaction sequence has been applied in the total syntheses of dipeptides [44], (+)-pilocarpine [45], (-)-kainic acid [46] and pipecolates such as (+)-vincamines [20], for example. Also achiral compounds, such as prodigiosin [47] have been synthesized using the Pf-protecting group.

Variously functionalized L-asparagine derivatives have been used as starting materials in particularly many cases. It is common that alkylation is followed by ester hydrolysis, which has often turned out to be problematic. For example, Pf is cleaved by the usual acidic ester hydrolysis. For this reason Pf is typically used only in the early steps to direct enolization and to avoid racemization. After the critical phase it is changed to a more familiar protecting group such as Boc.

### 9.1. (+)-Vincamine

**Scheme 32:**
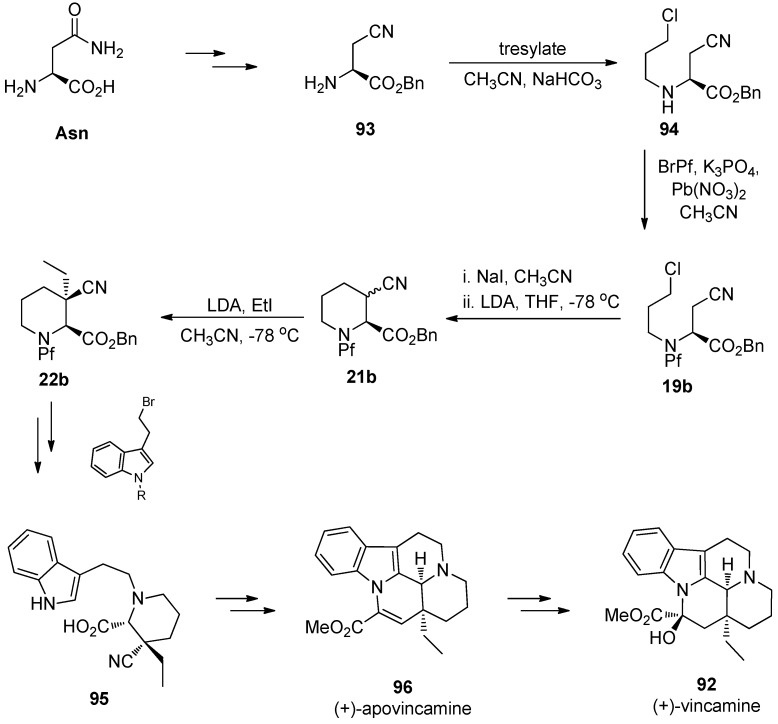
The synthesis of (+)-vincamine.

The synthesis of the pipecolate structure **22b** is one of the first examples in which Pf has been used as a protecting group [3]. Regioselective enolization was unsuccessful with carbamate protection, therefore trityl protection was investigated. However, trityl amines were not usable because of their acid lability, and that is why Pf-protection was tried. The pipecolate structure has been used in the total syntheses of apovincamine [3] and later vincamine [20]. 

(+)-Vincamine (**92**), an alkaloid that has an influence on brain’s blood circulation, is used in nootropic memory enhancing purposes. There are several routes to racemic vincamine, and it can be synthesized enantiopure from L-asparagine [20]. The details of the synthesis of pipecolates was presented in Section 4.2 (synthesis of piperidine and alkylation of pipecolate) (Scheme 32). 

### 9.2. (+)-Pilocarpine

Pilocarpine (**97**), an alkaloid isolated from tropical American *Pilocarpus* shrubs, is a muscarinic receptor agonist. It is used as a parasympathomamimetic compound towards glaucoma and xerostomia. There are several syntheses for racemic pilocarpine, and it can be made enantiopure from L-asparagine [45]. *N*-Pf-Protected asparagine **18a** is alkylated, converted from amino acid to bromo acid to finally form an α,β-disubstituted succinate **98** and further the product.

**Scheme 33:**
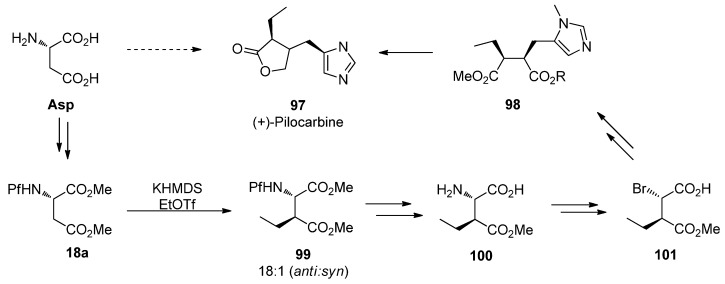
The synthesis of (+)-pilocarpine.

### 9.3. (-)-Kainic acid

Kainic acid (**102**) is a marine natural product belonging to the group of kainoids, glutamate analogs, that exhibits a wide range of biological activities. Kainoids are believed to have an influence on nervous system and they have been discovered to be useful also as insecticides. In this synthesis of kainic acid an inexpensive *trans-*hydroxy-L-proline is used as the starting material. The key step is diastereoselective alkylation of a keto derivative of *N*-Pf-hydroxyproline **33**, which is followed by stereoselective reduction of the keto function. In this synthesis, Pf is used solely in these steps and then replaced with a benzyloxycarbonyl (Cbz) in order to facilitate the upcoming cuprate substitution, which ultimately leads to the desired product [46].

**Scheme 34:**
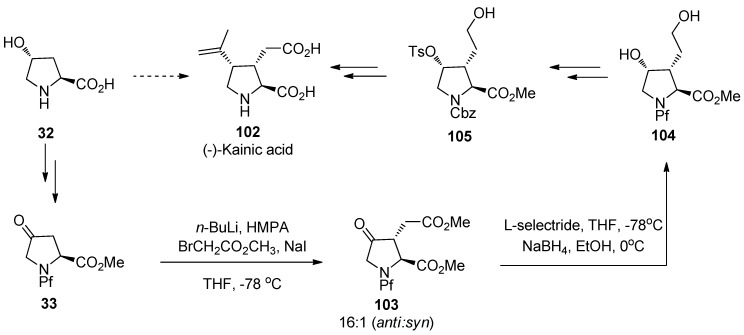
The synthesis of (-)-kainic acid.

### 9.4. γ-Lactams from dipeptides

Lactams are conformational constraints in peptide skeletons and are effective structural tools for probing the active conformation of bioactive peptides. In the same way, altering their structure gives more information about how the conformation affects on biological activity.

**Scheme 35:**
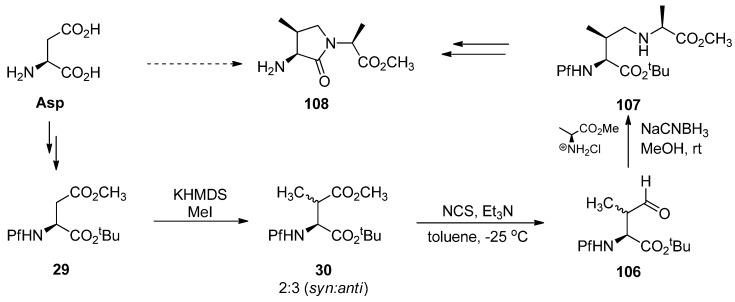
Synthesis of γ-lactam dipeptides.

Different kinds of dipeptides leading to γ-lactams have been made enantiopure from *N*-Pf-L-asparagine. Synthesis exploits the well-known feature of Pf of preventing enolization at the α-carbon of this diester **29 **that enables regioselective alkylation. It also protects the α-*tert*-butyl ester from reduction. Pf-protection is, however, changed after it had served its above-mentioned function because it does not tolerate the acidic conditions needed to cleave *tert*-butyl ester [44]. 

## 10. Phenylfluorenyl Derivatives

**Figure 14:**
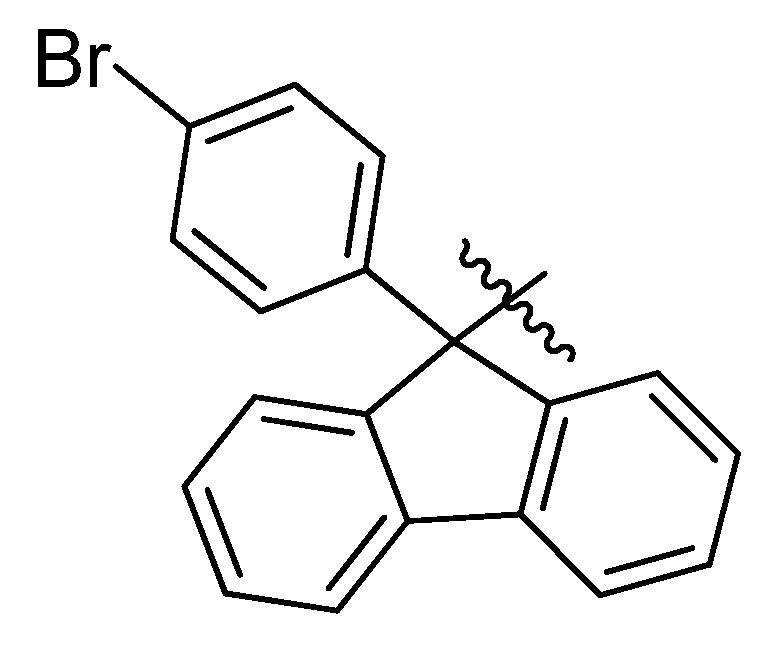
*p*-Bromophenylfluorenyl.

Modifications of the core structure of Pf, and how they might effect on the characteristics of Pf as a protecting group, have not been studied. Different halogen-, alkyl- or aryl-substituted fluorenyl compounds have been made and examined in different contexts [48,49,50], but using Pf-related structures as protecting groups for nitrogen is very rare. The only known Pf-derivative used in synthesis is *p*-bromo phenylfluorenyl (BrPf) (Figure 14), that has been made to make it easier to remove Pf under mildly acidic conditions [51].

**Scheme 36:**
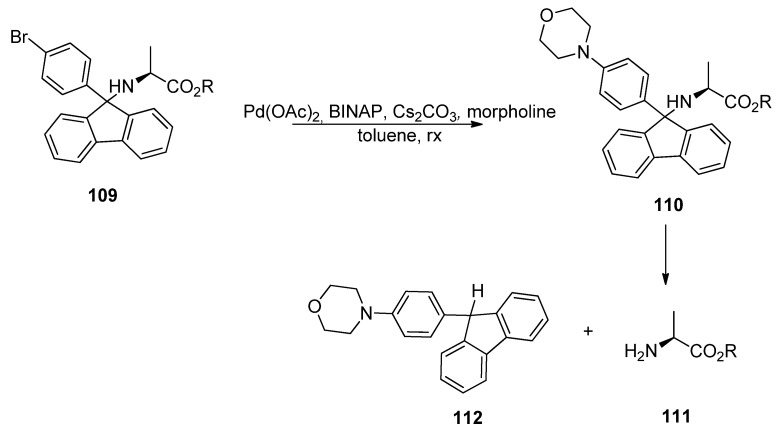
Palladium-catalyzed cross-coupling in the removal of BrPf.

The removal of BrPf has two phases. First, BrPf is activated by palladium-catalyzed amination between the aryl bromine and morpholine. The *p*-aminophenylfluorenyl compound that is formed forms a more stable cation than Pf-cation, and it can be removed under mildly acidic conditions. It can also be removed selectively in the presence of *tert*-butyl esters and carbamates. This works also the other way around, so that *tert*-butyl esters and carbamates can be removed selectively in the presence of BrPf (Scheme 36) [51].

## 11. Phenylfluorenyl on Solid Support

Solid phase organic synthesis is a useful tool for making large libraries of molecules. The substrate is temporarily bound to a polymeric resin, which is removed at the end of the reaction sequence. The intermediate resin bound products can be filtered from the solutions and there is no need for a separate purification. Reactions are usually driven to completion by using excess reagents. Slowness is the most common disadvantage of heterogenic reactions [52]. 

Synthetic strategies based on solid supported reagents are also becoming more common in the synthesis of small molecules. There is a so called linker between the polymer and the first building block, to which the molecule is attached and by the help of which the polymer can be removed. There exist several different linkers to amines, but they are usually sensitive to strong bases, acids or conditions in organometallic reactions, which constricts their use. Trityl linkers have been used to attach amines, carboxylic acids and alcohols.

**Scheme 37:**
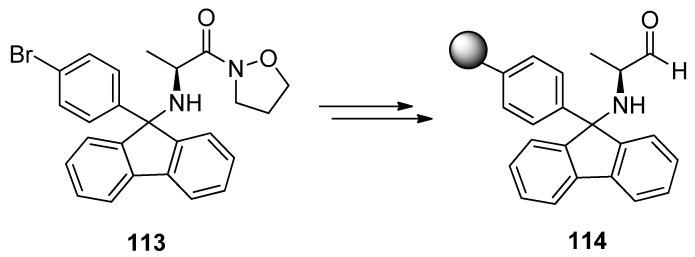
Pf-linker from BrPf.

Pf as a protecting group for α-amino carbonyls has proven its utility in liquid phase synthesis. Its possibilities in solid phase synthesis have been examined [53,54,55] and it can act as a linker in the syntheses of alkaloids, heterocycles and amino acids. Structurally stable Pf-protected amino compounds on solid support are a valuable source in the building of chiral amino compounds libraries. Pf-linker can be made from BrPf, for example (Scheme 37).

## 12. Conclusions

The phenylfluorenyl group has been proven to reliably protect the α-center in amino carbonyl compounds against epimerisation. It is especially useful in regioselective reactions such as enolization and alkylation. Despiet its steric bulk, it does not seem to have a significant influence on the diastereoselectivity of these or any other reactions. Very little is actually known about the Pf groups real steric and/or electronic effects or possibilities in synthesis. Much remains to be explored, especially to learn more about the characteristics of its derivatives in terms of stability, diastereoselectivity and the protection of the amine itself. At this point the Pf group is, though, unique in its way to allow a chemist to efficiently protect the usually stereochemically vulnerable α-center of α-amino acids and ketones against epimerization. 
